# The Role of Zinc in the Pathogenesis of Lung Disease

**DOI:** 10.3390/nu14102115

**Published:** 2022-05-19

**Authors:** Xiaoying Liu, Md Khadem Ali, Kamal Dua, Ran Xu

**Affiliations:** 1 Department of Breast Surgery, The First Affiliated Hospital of China Medical University, Shenyang 110001, China; xiaoyingliu@cmu.edu.cn; 2 Division of Pulmonary and Critical Care Medicine, School of Medicine, Stanford University, Stanford, CA 94305, USA; mdali@stanford.edu; 3 Vera Moulton Wall Center for Pulmonary Vascular Disease, Stanford University, Stanford, CA 94305, USA; 4 Discipline of Pharmacy, Graduate School of Health, University of Technology Sydney, Sydney, NSW 2007, Australia; kamal.dua@uts.edu.au; 5 Faculty of Health, Australian Research Centre in Complementary and Integrative Medicine, University of Technology Sydney, Ultimo, NSW 2007, Australia; 6 Uttaranchal Institute of Pharmaceutical Sciences, Uttaranchal University, Dehradun 248007, India; 7 Department of Thoracic Surgery, Shengjing Hospital, China Medical University, Shenyang 110022, China

**Keywords:** zinc, COPD, asthma, cystic fibrosis, pulmonary fibrosis, pulmonary hypertension

## Abstract

Lung diseases, such as asthma, chronic obstructive pulmonary diseases (COPD), and cystic fibrosis (CF), are among the leading causes of mortality and morbidity globally. They contribute to substantial economic burdens on society and individuals. Currently, only a few treatments are available to slow the development and progression of these diseases. Thus, there is an urgent unmet need to develop effective therapies to improve quality of life and limit healthcare costs. An increasing body of clinical and experimental evidence suggests that altered zinc and its regulatory protein levels in the systemic circulation and in the lungs are associated with these disease’s development and progression. Zinc plays a crucial role in human enzyme activity, making it an essential trace element. As a cofactor in metalloenzymes and metalloproteins, zinc involves a wide range of biological processes, such as gene transcription, translation, phagocytosis, and immunoglobulin and cytokine production in both health and disease. Zinc has gained considerable interest in these lung diseases because of its anti-inflammatory, antioxidant, immune, and metabolic modulatory properties. Here we highlight the role and mechanisms of zinc in the pathogenesis of asthma, COPD, CF, acute respiratory distress syndrome, idiopathic pulmonary fibrosis, and pulmonary hypertension.

## 1. Background

Zinc is a critical nutritional trace element required for the growth and development of almost all known organisms. Zinc plays crucial roles in maintaining cellular, molecular, and systemic biological processes such as cell proliferation, differentiation, apoptosis, DNA and RNA synthesis, RBC production, tissue maintenance, immune function, glucose and lipid metabolism, and cell signaling pathways [1,2,3]. Zinc is required for the function and structure of about 2500 proteins, accounting for about 10% of all human proteins, including important enzymes, cytokines, transcription factors, and other proteins [4]. Zinc acts as a structural, catalytic, and regulatory signaling molecule. About 1.4–2.3 g of zinc is found in a healthy human adult, 85% of which resides in muscle tissues and bones, 11% in skin, and 4% in all other tissues. In plasma or serum, zinc exists as bound to albumin (60%), alpha-macroglobulin (30%), and transferrin (10%). Under normal physiological conditions, zinc level is tightly regulated in the body through a coordinated and combined carrier-mediated mechanism during absorption, transport, and secretion processes. Zinc is primarily taken up in the small intestine from foods. The efficiency of zinc absorption is influenced by several factors, including zinc status in the body and zinc concentration in the diet. The zinc “importers” ZIP 1–14, the zinc exporters ZnT 1–10, and numerous binding proteins maintain zinc homeostasis in the body [5]. 

**Figure 1:**
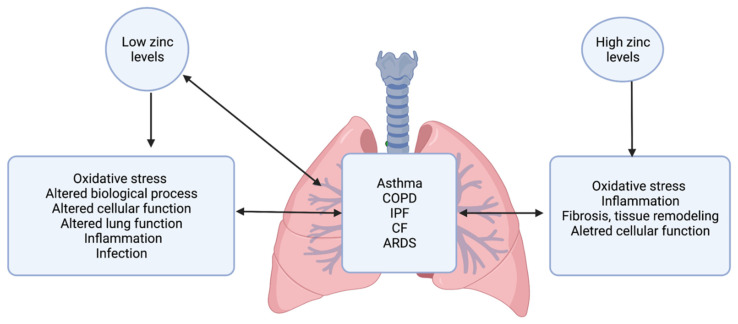
Role of zinc dysregulation in lung disease.

Zinc has long been seen as a “two-edged sword” in the body, as both too high and too low zinc levels have been related to negative consequences in cells and tissues. There has been evidence of low zinc levels in a number of conditions, including asthma, lung cancer, diabetes, hypertension, and autoimmune and other inflammatory diseases [6,7,8,9]. Low zinc levels promote apoptosis in systemic endothelium and respiratory epithelium, while high zinc levels can also induce apoptosis in cultured cerebral and pulmonary endothelia [10]. On the other hand, low zinc levels have been demonstrated to prevent apoptosis in systemic endothelial cells triggered by cadmium, linoleic acid, and tumor necrosis factor alpha [10]. High zinc levels can also increase the production of reactive oxygen species, which induces oxidative stress in the lungs. Many epidemiological, clinical, and experimental evidence suggests that aberrant levels of zinc and zinc regulatory proteins are associated with the development and/or progression of several lung diseases, including asthma, chronic obstructive pulmonary disease (COPD), cystic fibrosis (CF), idiopathic pulmonary fibrosis (IPF), coronavirus disease 2019 (COVID-19), acute respiratory distress syndrome (ARDS), pulmonary hypertension (PH), and respiratory infectious diseases (Figure 1, Table 1). Several state-of-the-art reviews have already summarized current knowledge of the role of zinc and zinc-regulatory molecules in COVID-19 [11,12,13,14,15] and different respiratory tract infections [16,17]. Thus, in this review, we do not focus on COVID-19 and different respiratory tract infections; instead, we highlight current knowledge of the role of zinc and zinc-regulatory proteins in asthma, COPD, IPF, CF, ARDS, and PH.

## 2. Regulation of Zinc Levels in the Lung

The respiratory epithelium is now widely recognized as the lung’s first line of defense against a wide range of exogenous and endogenous insults. In the respiratory epithelium, zinc plays an important role in preventing death receptor–mediated apoptosis and barrier failure. Zinc can also function as an anti-inflammatory, an antioxidant, an anti-apoptotic molecule, an organelle stabilizer, a critical wound healing component, and a cofactor for DNA synthesis in respiratory epithelium [38]. Bao and Knoell demonstrated that zinc depletion increases apoptosis and causes degradation of junction proteins, loss of cell-to-cell contact, and enhanced epithelial permeability [39]. In a study of rats fed a zinc-deficient diet, the diet was shown to decrease bronchial cilia length, number of cilia, and number of cilia per cell, while zinc supplementation affected the integrity of the bronchial epithelium. Apart from airway epithelial cells, zinc is also known to exert immunomodulatory effects and antimicrobial effects on monocytes, including alveolar macrophages [40]. Human monocytes and airway epithelium have been reported to have an important role for zinc, ZIP8, and NFkB [41]. Chronic alcohol intake has been shown to reduce zinc levels and the functionality of alveolar macrophages, particularly phagocytic activity, in humans and rats [42,43].

## 3. The Role of Zinc Dyshomeostasis in Lung Disease

### 3.1. Asthma

Asthma is an inflammatory disease of the airways and lungs that can develop at any age and affects approximately 10% of the population in developed nations [44]. The disease is characterized by aberrant responses to otherwise harmless environmental stimuli, causing chronic inflammation, smooth muscle contraction, and mucus hypersecretion in the airways. This results in airway narrowing, which leads to airflow obstruction and breathing difficulties [44]. Numerous studies have demonstrated a significant decrease in zinc levels in the serum of asthma patients [18,45,46,47,48,49,50,51,52,53]. However, some studies did not find this decrease in serum zinc levels in asthma patients [54,55,56,57]. Zinc levels were also low in the sputum [58], saliva [59], and nails [60], but not in the erythrocytes [61], of asthmatic patients. An asthma-related symptom, wheezing, was linked with low zinc content [62,63,64]. There was also a significant negative relationship between serum zinc levels and total IgE and a positive relationship between zinc levels and FEV1 [18]. In a murine model of ovalbumin-induced allergic airway inflammation in the context of low zinc condition, zinc deficiency was shown to induce oxidative damage and airway epithelial cell apoptosis [19]. Mice fed a zinc-deficient diet had worse asthma symptoms, such as increased airway hyper-responsiveness, airway inflammation, and epithelial apoptosis, compared to mice fed a normal diet [19]. 

An appropriate zinc level in the body is crucial for maintaining the balance between anti- and pro-oxidative and anti- and pro-inflammatory responses that are disrupted in asthma. As low zinc levels have been linked to asthma in preclinical studies, zinc supplementation or the promotion of zinc homeostasis would be a new treatment option for asthma patients. The researchers found that zinc supplementation with drinking water reduced the number of eosinophils in the bronchoalveolar lavage fluid (BALF) of mice treated with ovalbumin [65]. Zinc supplementation has been reported to improve asthma symptoms in asthma patients [20]. In addition, appropriate zinc consumption during pregnancy was linked with a reduced risk of wheezing among newborns [66]. Zinc supplementation by inhalation, however, may lead to allergic inflammation. For instance, in allergic and non-allergic mouse models, ZnoNP increased airway inflammation [67,68]. Zinc supplementation for asthma needs further evaluation and optimization.

### 3.2. COPD

COPD is one of the leading causes of mortality and morbidity globally, with a significant social and economic burden [69,70,71,72]. COPD is a complex disease of the airways and lungs characterized by chronic airway inflammation associated with alveolar destruction (i.e., emphysema), airway remodeling, and irreversible airflow limitation. The most common cause of COPD is cigarette smoking; however, nonsmokers can also develop the disease. Currently, COPD is incurable, but some treatments can reduce the symptoms and slow the progression of the disease.

Many clinical and experimental studies have shown that dysregulation of zinc and zinc-associated proteins is linked with COPD pathogenesis [22,24,73,74,75,76,77]. Clinically, smokers with low dietary zinc intake exhibit a significantly higher prevalence of COPD [73]. Notably, insufficient dietary zinc intake was found to be common in patients with COPD [74,75,76]. However, it is unclear how insufficient dietary zinc intake leads to pulmonary dysfunction in smokers and COPD. There have also been numerous studies demonstrating that low zinc levels associated with COPD and cigarette smoke are linked with airflow obstruction, oxidative stress, inflammation, apoptosis, DNA damage, and risk of infections, allergens, and cancer [73,76,78,79,80,81,82,83]. Moreover, randomized prospective clinical trials of dietary and oral nutritional supplements improved several clinically relevant functional outcomes, such as total intake, anthropometric measures, grip strength, inspiratory and expiratory muscle strength, and exercise performance in patients with COPD [84,85]. These findings suggest that nutritional supplements may be effective in treating COPD. 

A study recently showed a high incidence of low zinc levels in forty-one rural Midwestern veterans with COPD and agricultural dust exposures [21]. This study also showed that zinc-deficient mice show more severe airway inflammation in response to repeated agriculture dust exposure for 21 days. Likewise, to determine the effect of low zinc intake on chronic cigarette smoke (CS) exposure–induced experimental COPD, Knoell et al. conducted experiments on mice fed a moderate zinc-deficient diet or transgenic Zip8 knockout and overexpressing mice exposed to either room air or cigarette smoke [22]. The authors showed that mice fed a restricted zinc diet have significantly increased CS-induced emphysema-like alveolar enlargement compared to mice fed a normal diet. Additionally, even when mice were given the normal zinc diet, Zip8 depletion or overexpression showed worse lung damage in mice exposed to chronic CS exposure. The results from this study support the idea of developing micronutrient-based therapies that can boost zinc levels and identify a vital zinc transporter, Zip8, that is important in maintaining balance within the lung microenvironment by protecting it from CS-induced lung damage. However, it is unclear from the study how increased and decreased Zip8 expression induced the prolonged CS-induced alveolar tissue loss. In the future, it would be critical to determine how zip8 and zinc dyshomeostasis affects lung cellular functions, inflammation, ROS production, and chemokine production in the context of CS exposure.

Hamon et al. measured zinc concentrations in the bronchoalveolar lavage (BAL) supernatant of 20 healthy controls, 17 healthy smokers, and 20 current and 19 ex-smoker COPD subjects [23]. The authors showed that zinc concentrations in the bronchoalveolar lavage fluid of smokers and patients with COPD are significantly decreased and positively associated with alveolar macrophage efferocytosis [23], a process by which phagocytic cells remove apoptotic cells. Further in vitro studies revealed that zinc chelator TPEN treatment significantly decreased efferocytosis in macrophages. The authors then showed a significant reduction in efferocytosis ability and intracellular zinc levels in the macrophages isolated from zinc transporter Zip1 null mice, indicating that ZIP1 could play a critical role in the macrophage efferocytosis process. They also demonstrated that zinc chelation to mimic zinc deficiency increases ZIP2 expression while no change took place in ZIP1 expression in the human THP-1 macrophage cell line, suggesting that in macrophages, zinc homeostasis is maintained by zinc transporters ZIP1 and ZIP2 differently responded to zinc deficiency. Moreover, using a CS-induced mouse model of COPD, a human ex vivo air–liquid interface model, and human lung tissues from COPD patients with and without a smoking history, the same research team also demonstrated an uncoupling role of zinc trafficking and autophagy in airway epithelial cells that play a vital role and that could be a therapeutic target in COPD [24].

### 3.3. CF

CF is a chronic and progressive genetic disease linked with mutations in the CFTR gene and defective chloride transport across the epithelial cell membranes. The clinical symptoms of CF include a thick, dry mucus that obstructs the airways, persistent pulmonary infections, bronchiectasis, pancreatic insufficiency, and increased chloride levels in sweat.

Several studies have shown a dysregulation of zinc levels (lower in blood and higher in sputum) in adults and children with CF [25,58,86,87,88,89]. In the first three years of life, low serum zinc contents were detected in one-third of children with CF [86]. This study also found a discrepant link between growth and serum zinc in cross-sectional and longitudinal analyses; further study to better understand the role of zinc in growth in children with CF is warranted. A low-plasma zinc concentration was reported in adults with CF and moderate lung disease who had good nutritional status, and that low zinc level was linked with worse clinical outcomes [25]. There is variable success with oral zinc supplements in CF [90,91,92]. A recent exciting study showed that zinc deficiency through the unique splicing switch of ZIP2 contributes to CF-associated MUC5AC hypersecretion in airway epithelial cells [26]. Importantly, evidence from both in vivo and in vitro experiments of the CF model suggests that mucosal iron supplementation can restore airway epithelial cells’ chloride secretion by stimulating calcium-dependent chloride channels [27]. In addition to the potential therapeutic value of this finding, if zinc supplements have to be applied to mucosal surfaces of the airways to be effective, it would be imperative to develop efficient and safe methods of delivering zinc into the airways. While zinc is a relatively non-toxic metal when administered as an oral supplement, its direct administration into the airways can cause several side effects, including olfactory loss and inflammation of the respiratory tract accompanied by bronchial hyperresponsiveness [93,94]. Additionally, several recent reports showed that airborne particulate matter contains significant quantities of zinc, leading to sensitization to common aeroallergens and the occurrence or exacerbation of respiratory or allergic conditions [95,96,97].

### 3.4. IPF

Although extensive research has been conducted on pulmonary fibrosis, how pulmonary fibrosis occurs remains largely unclear, which has caused the unfortunate lack of effective treatments for patients suffering from progressive pulmonary fibrosis. IPF is a chronic and most common form of interstitial lung disease. The etiology and pathogenesis of IPF are unknown. IPF is characterized by repeated epithelial cell injuries and insufficient alveolar epithelium repair, which leads to excessive fibroblast activation and lung fibrosis [98,99]. The clinical symptoms of IPF include cough, dyspnea, and increasing immobility [100]. IPF primarily affects patients over the age of 60. It has a poor prognosis, with a median survival time ranging between 3 and 5 years [100,101,102]. The incidence rate for IPF has greatly differed by geography; North America and eastern Europe have experienced the highest prevalence, averaging between 3 and 9 new cases per 100,000 person-years, while East Asia had the lowest, averaging fewer than 4 new cases per 100,000 person-years [103,104]. While the etiology is unknown, several risk factors may contribute to the onset and progression of IPF, such as aging, air pollution, smoking habits, microbial infections, and occupational exposure [102,105]. 

Several experimental studies have reported evidence that altered lung and systemic levels of zinc and zinc-regulatory molecules are associated with pulmonary fibrosis [28,29,30,97,106,107,108]. Zinc-deficiency exacerbated ventilation-induced lung damage in mice and rats [28,29]. Furthermore, Zhang et al. demonstrated that mice fed a zinc-deficient diet have increased oxidative stress and inflammation, a reduced activity of antioxidant enzymes, and a subsequently induced fibrosis in the lung compared to mice fed a normal diet [30]. A very recent study of single-cell RNA-seq analysis of epithelial cells collected from patients with IPF and aged injured mice identified a zinc–metabolic defect of alveolar progenitor cells (AEC2). A significant decrease in levels of the specific zinc transporter ZIP8 was shown in the AEC2. The authors also showed that AEC2-specific deletion of Zip8 in mice and the feeding of a low-zinc-content diet to mice leads to exacerbated bleomycin-induced lung fibrosis. These results suggest that zinc metabolism and the zinc transporter ZIP8 play critical roles in regulating alveolar progenitor renewal. During aging and in IPF, the zinc transporter ZIP8 of AEC2s is reduced, leading to impaired alveolar repair and causing pulmonary fibrosis.

Moreover, zinc in the particulate matter of atmospheric dust samples and zinc salts has been shown to be toxic in the lung of mice [97]. Importantly, this study revealed that after four weeks of zinc salts administration in mice, the mice showed significant induction of inflammation and fibrosis in the lung [97], suggesting that inhaling particulates that contain a high soluble metal content, like zinc, may have a crucial effect on the pulmonary cells. In addition, zinc oxide nanoparticles (ZnoNP) are widely used in different commercial products, such as food additives, personal hygiene products, paints, cosmetics, and textiles. ZnoNP prompted the proliferation of airway epithelial cells and pulmonary fibrosis in mice [108]. Another study by Wang et al. demonstrated that compared with controls, ZnoNP-exposed mice showed significantly reduced body weight and increased total protein, hydroxyproline content, and total cell numbers in their bronchoalveolar lavage fluid and malondialdehyde and nitric oxide levels in the lung [109]. The authors also found hyperplastic changes and inflammation in the lungs of the ZnoNP-exposed mice.

Furthermore, He et al. uncovered a novel mechanism of Cu, Zn-SOD-mediated, and Th2-independent M2 macrophage polarization and provided a potential therapeutic target for attenuating the promoted development of lung fibrosis [106]. These findings suggest that both zinc deficiency and toxicity can cause lung damage. Zinc levels in the body need to be tightly regulated to maintain homeostasis and prevent the occurrence and progression of diseases like pulmonary fibrosis.

### 3.5. ARDS

ARDS is a serious, life-threatening lung disease associated with various etiologies either through indirect or direct lung insults and is characterized by moderate-to-severe hypoxemia, abnormal respiratory system compliance, and acute pulmonary infiltrates. Clinical symptoms include shortness of breath, breathing difficulties, dizziness, and confusion. Previous studies showed significant down-regulation of zinc levels in the plasma of patients with ARDS compared to healthy controls [28,110]. Gonçalves et al. analyzed serum zinc levels of 269 critically ill patients infected by severe acute respiratory syndrome coronavirus 2 and correlated with its association with ARDS. The authors found that low serum levels and a high prevalence of low-serum zinc levels were associated with severe ARDS [31]. Zinc deficiency in alveolar macrophages and lung epitheliums can decrease lung barrier function, leading to respiratory distress syndrome [32]. In the lung of a murine sepsis model, zinc deficiency increased lung and other organ damage [33]. Gomez et al. found that lipid concentration, especially the phospholipids, is altered in the lungs of rats fed a zinc-deficient diet [111].

In contrast, inhalation of zinc fumes has been shown to cause ARDS in mice. However, the underlying mechanism(s) of how zinc deficiency induces lung injury in the context of ARDS is unclear. Future studies are needed to explore the potential mechanisms of how altered zinc homeostasis causes ARDS and whether dysregulation of zinc could be therapeutically targeted in the disease. 

### 3.6. PH

PH is a complex vascular disease characterized by abnormally high pressure in the blood vessels that affects the arteries in the lungs and the right side of the heart. Based on their causes, there are five different groups of PH: pulmonary arterial hypertension (PAH), PH due to left heart disease, PH due to chronic lung disease (e.g., COPD, interstitial lung disease, CF) and hypoxia, PH due to chronic blood clots in the lungs, and PH due to unknown causes. There is no cure for PH, but there are treatments available to help improve symptoms and slow the progression of the disease. Thus, there is an urgent unmet need to develop effective therapies to treat the disease. 

Emerging evidence suggests that altered zinc and zinc transporter protein levels are associated with the occurrence and progression of PH [34,35,36,37]. A previous study by Zhao et al. identified that plasmalemma zinc transporter ZIP12, encoded by the gene Slc39a12, plays a critical role in hypoxia-induced pulmonary vascular remodeling [34]. ZIP12 expression was shown to be up-regulated in remodeled pulmonary vascular tissues of humans, rats, and cows susceptible to hypoxia-induced PH. A significant up-regulation of ZIP12 was also found in the lung of idiopathic PAH patients and monocrotaline-treated rats, another well-established PH animal model. Further in vitro studies showed that siRNA-mediated inhibition of ZIP12 diminished hypoxia-induced increases in intracellular labile zinc contents and proliferation of pulmonary arterial smooth muscle cells (PASMC). Zip12 genetic disruption inhibited hypoxia-induced PH in rats, as evidenced by a significant decrease in pulmonary arterial pressure, right heart hypertrophy, and muscularization. While the results of these studies point to ZIP12 having a fundamental role in regulating pulmonary vascular homeostasis and remodeling under hypoxic conditions, the underlying mechanisms of how ZIP12 affects hypoxic responses remain unclear. Another study by Xiao et al. demonstrated that increased intracellular labile zinc, possibly from ZIP12, was linked with reduced phosphatases, increased transcription factor CREB-mediated activity, and PASMC proliferation [35]. Zhu et al. showed that ZIP12 contributed to phenotypic switching in hypoxia-induced PASMCs and promoted PH. The authors proposed that the HIF-1/ZIP12/pERK-signaling axis could facilitate hypoxia-induced phenotypic switching in PASMCs [36]. A very recent study showed that aberrant levels of zinc homeostasis (ZIP12, MT3), S1P signaling (S1PRs, SPNS2), and vascular remodeling (αSMA, FI, RVSP) are associated with each other in the monocrotaline-induced PH rat model, and BMPR2-targeted therapy might alleviate this condition [37]. Together, these findings suggest a new therapeutic avenue for preventing or treating PH by inhibiting ZIP12 and suppressing excursions of intracellular free zinc.

## 4. Conclusions and Future Roadmap

Emerging evidence has shown that zinc is critical for the homeostasis of multiple organs and systems at the cellular, molecular, and systemic levels. Zinc dyshomeostasis has been linked with several lung diseases, such as asthma, COPD, IPF, and CF. Currently, it largely remains unclear how altered zinc and zinc regulatory molecules contribute to the development and progression of lung disease. There are still several questions about zinc dyshomeostasis and lung diseases. Is a low zinc level a major or minor contributing factor in the development and progression of lung diseases? Are low zinc levels the cause or the result of lung diseases? Could zinc supplementation be effective as a treatment for each of these disorders if zinc insufficiency is the cause (Table 2)? Or would alternative measures, such as controlling the expression of zinc transporters and developing more targeted therapies, be required to restore zinc homeostasis and improve disease outcomes? More research on zinc homeostasis is needed to answer these questions, which could pave the way for new therapeutics targeting zinc homeostasis in lung inflammatory disorders.

## Figures and Tables

**Table 1:** Role of zinc and zinc-regulatory molecules in lung disease.

Disease	Zinc Status	Primary outcome	Mechanism	Reference
Asthma	Low serum zinc levels	Serum zinc levels correlated with total IgE levels and forced expiratory volume in the first second (FEV1).	Zinc triggered IFN-γ and inhibited IL-10 production in PBMCs of asthmatics	[18]
	Low zinc diet	Mice fed with a low zinc diet had greater levels of airway hyper-responsiveness (AHR), eosinophilia, and mucus cell hyperplasia, increased active caspase-3 and apoptosis	-	[19]
	Zinc supplementation	Zinc supplementations improved asthma symptoms in asthma patients.	-	[20]
COPD	Zinc deficient diet	Zinc-deficient mice showed more severe airway inflammation in response to repeated agriculture dust exposure.	-	[21]
	Zinc deficient diet, ZIP8 KO	Mice fed a restricted zinc diet had significantly increased CS-induced emphysema and ROS formation in the lung; Zip8 KO depletion or overexpression showed worse lung damage in mice exposed to chronic CS exposure.	-	[22]
	Low zinc levels in bronchoalveolar lavage fluid (BALF) of COPD patients.Zinc chelator	Zinc levels in BALF positively correlated with alveolar macrophage efferocytosis. TPEN significantly decreased efferocytosis in macrophages. Zinc transporters ZIP1 and ZIP2 differently responded to zinc-deficiency.	-	[23]
	-	Uncoupling role of zinc trafficking and autophagy in airway epithelial cells	-	[24]
CF	Low plasma zinc in adult CF patients	Low zinc level was linked with worse clinical outcomes.	-	[25]
	Low zinc levels due to ZIP2 splicing switch	Zinc deficiency contributed to CF-associated MUC5AC hypersecretion in airway epithelial cells.	-	[26]
	Extracellular zinc supplementation	Extracellular zinc and ATP restored impaired chloride secretion in CF airway epithelium.	Through stimulating calcium-dependent chloride channels	[27]
IPF	Low zinc diet	Zinc deficiency exacerbated ventilation-induced lung damage in mice and rats.	-	[28,29]
	Low zinc diet	Mice fed a zinc-deficient diet had increased oxidative stress and inflammation, reduced activity of antioxidant enzymes, and subsequently induced fibrosis in the lung	-	[30]
	ZIP8 KO, low zinc diet	AEC2-specific deletion of Zip8 in mice and mice fed a low zinc content diet has exacerbated bleomycin-induced lung fibrosis.	-	-
ARDS	Low serum zinc of ARDS patients	low serum levels and a high prevalence of low serum zinc levels were associated with severe ARDS	-	[31].
	zinc deficiency	Zinc deficiency in alveolar macrophages and lung epitheliums decreased lung barrier function, leading to ARDS	-	[32].
	Zinc deficiency	zinc deficiency induced lung and other organ damage	-	[33]
	Zinc fume inhalation	Zinc fume developed ARDS in mice	-	
PH	ZIP12 upregulation in lung of IPAH patients, animal models	ZIP12 knockdown diminished hypoxia-induced increases in intracellular labile Zinc contents and proliferation of PASMC. Zip12 KO inhibited hypoxia-induced PH in rats, as evidenced by a significant decrease in pulmonary arterial pressure, right heart hypertrophy, and muscularization.		[34]
	High intracellular zinc, ZIP12 upregulation	Increased intracellular labile zinc, possibly from ZIP12, was linked with reduced phosphatases, increased transcription factor CREB-mediated activity, and PASMC proliferation		[35]
	ZIP12 upregulation	ZIP12 contributed to hypoxia induced PASMCs phenotypic switch and promoted PH.	HIF-1/ZIP12/pERK signaling axis could facilitate hypoxia-induced phenotypic switching in PASMCs	[36]
	Altered zinc homeostasis	Altered levels of zinc homeostasis (ZIP12, MT3), S1P signaling (S1PRs, SPNS2), and vascular remodeling (αSMA, FI, RVSP) were associated with each other in the monocrotaline-induced PH rat model	-	[37]

**Table 2:** Clinical trials that study zinc supplementation in lung disease.

Disease	Clinicaltrial.gov ID	Study Design	Participants	Study Duration	Intervention Nutrient with Dosage	Primary Outcome	Key Findings	Ref
Asthma	TCTR20141212001	Double blinded RCT	42	12 months	zinc bis-glycinate (30 mg elemental zinc/day)	Pediatric respiratory assessment measure (PRAM)	PRAM score decreased at 24 and 48 h	[20]
	-	Double-blind, randomized, placebo-controlled clinical trial	284	8 weeks	Zinc supplements (50 mg/day)	Zinc balance and asthma clinical symptoms	Significantly improved zinc levels; clinical symptoms such as cough, wheezing, and dyspnoea; and lung function parameters (FVC, FEV1 and FEV1/FVC.	[112]
CF	NCT00104494	Randomized, Parallel Assignment	30	8 weeks	Zinc acetate (20 mg/day)	Zinc balance	-	-
	-	Double blind placebo-controlled pilot study	26	12 months	Zinc (30 mg/day)	Rate of respiratory tract infections, antibiotics use, plasma cytokines	Reduced the number of days of oral antibiotics used to treat RTIs in children with CF	[91]
	CTRI/2011/12/002230	Double-blind randomized placebo-controlled trial	40	12 months	Zinc tablets (30 mg/day)	A reduction in the average days of systemic antibiotics	Zinc supplementation did not reduce lung infection in children with CF	[92]
COPD	-	Randomized controlled trial	30	8 weeks	Zinc picolinate (22 mg/day)	Oxidant stress, and pulmonary function	Favorable effects on oxidant–antioxidant balance	[81]
	-	Double blinded RCT	120	-	Sodium (100 mg/day); zinc (2 mg/day); and manganese (0.4 mg/day)	Effect of trace elements (Na, Mg, Zn) supplementation on the period the COPD patients spend on mechanical ventilation	The nutrition supplementation significantly reduced the period the patients with COPD spent on the mechanical ventilation	[78]

## Data Availability

Not applicable.
